# Investigating genome reduction of *Bordetella pertussis* using a multiplex PCR-based reverse line blot assay (mPCR/RLB)

**DOI:** 10.1186/1756-0500-7-727

**Published:** 2014-10-15

**Authors:** Connie Lam, Sophie Octavia, Vitali Sintchenko, Gwendolyn L Gilbert, Ruiting Lan

**Affiliations:** School of Biotechnology and Biomolecular Sciences, University of New South Wales, Sydney, NSW 2052 Australia; Centre for Infectious Diseases and Microbiology-Public Health, Institute of Clinical Pathology and Medical Research, Pathology-West, Westmead Hospital, Sydney, NSW 2145 Australia; Marie Bashir Institute for Emerging Infection and Biosecurity, Sydney Medical School, The University of Sydney, Sydney, NSW 2006 Australia

**Keywords:** *Bordetella pertussis* (*B. pertussis*), Reverse line blot (RLB), Genome reduction, Evolution

## Abstract

**Background:**

The genetic composition of the bacterium causing whooping cough, *Bordetella pertussis*, has been investigated using microarray studies in order to examine potential genetic contributors to the disease re-emergence in the past decade. Regions of difference (RDs) have been previously identified as clusters of genes flanked by insertion sequences which are variably present in different sets of isolates, and have also been shown to be potential markers of *B. pertussis* evolution.

This study used microarray data to identify and select a panel of RDs; primers and probes for these RDs were then designed to test for the presence or absence of these regions in a novel and less expensive multiplex PCR-based reverse line blot (mPCR/RLB) assay. By comparing the presence or absence of RDs, we aimed to determine the genomic variability of a diverse collection of *B. pertussis* strains and how they have changed over time.

**Results:**

A *B. pertussis* specific mPCR/RLB using 43 genes representing 30 RDs, was developed and used to characterise a set of 42 *B. pertussis* isolates. When mapped against the previously identified evolutionary relationships of the strains, the losses of two RDs - BP0910A - BP00930 and BP1948-BP1962 - were found to be associated with significant events in *B. pertussis* history: the loss of BP0910A - BP00930 coincided with introduction of whole cell vaccines in the 1950s while that of BP1948-BP1962 occurred after the introduction of acellular vaccines. The loss of BP1948-BP1962 also coincided with expansion of the most recent *B. pertussis* strains.

**Conclusions:**

The mPCR/RLB assay offers an inexpensive and fast method of determining the gene content of *B. pertussis* strains and also confirms that gene losses are an ongoing feature of *B. pertussis* evolution.

**Electronic supplementary material:**

The online version of this article (doi:10.1186/1756-0500-7-727) contains supplementary material, which is available to authorized users.

## Background

*Bordetella pertussis* is a highly homogenous organism which diverged from a *Bordetella bronchiseptica*-like ancestor 0.7-3.5 million years ago [1]. Although a high degree of sequence similarity exists between *B. pertussis*, *B. bronchiseptica* and *Bordetella parapertussis*, it has been recognised that significant losses of groups of genes, termed regions of difference (RDs), have occurred during *B. pertussis* evolution [2]. Comprehensive studies using comparative genome hybridisation (CGH) microarray experiments have investigated such changes to the *B. pertussis* genome and correlated the emergence of currently circulating isolates with the absence of specific RDs [3–5].

While CGH is a method that allows genome comparison amongst large numbers of isolates, it is costly and labour-intensive when screening large numbers of samples. In contrast, multiplex PCR (mPCR)-based reverse line blot (RLB) assay is an established method with diverse range of applications in pathogen detection and typing [6]. The RLB used in this assay is a DNA macroarray, which relies on attachment of biotinylated PCR-amplified products to specific amine-labeled DNA probes. The assay allows multiple isolates to be processed in a single blot and, by using a multiplex approach, more that 40 targets can be probed simultaneously without increasing the number of individual PCRs [6]. This technique has been successfully applied to genotyping of bacterial pathogens of public health importance, such as *Streptococcus agalactiae* [7], *Streptococcus pneumoniae* [8], uropathogenic *Escherichia coli* [9], *Staphylococcus aureus* [10], methicillin resistant *Staphylococcus aureus* [11] as well as different viral pathogens including human papilloma virus (HPV) [12] and human adenoviruses [13].

We previously used single nucleotide polymorphisms (SNPs) to characterise an international collection of over 300 *B. pertussis* isolates. These isolates were differentiated into 42 unique SNP profiles (SPs) and six distinct clusters (clusters I to VI) [14], with recently emerging isolates in cluster I. In this study, we used representative isolates of each of the 42 SPs to develop an inexpensive specific mPCR/RLB to further determine the genome variation in *B. pertussis*. The presence or absence of previously determined RDs as revealed by the mPCR/RLB was compared against and used in conjunction with previous SNP typing to observe genomic variability of *B. pertussis* through time.

## Results and discussion

### Development and application of multiplex PCR/ reverse line blot to detect selected regions of difference

**Figure 1:**
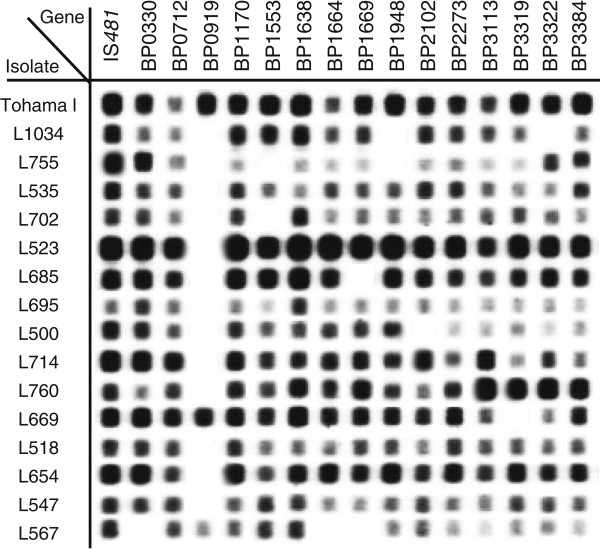
**Representation of an mPCR/RLB of 16 gene targets of 16** ***Bordetella pertussis*** **isolates.** Positive signals were interpreted as the presence of a gene, and no signal was interpreted as absent genes. IS*481* was used as a positive control for the 5-plex mPCR and Tohama I was used to ensure probe binding. The amplification of IS*481* and targets in Tohama I were always present. Genes that did not show a signal with Tohama I were excluded from analysis. If any isolate strain did not produce a positive signal for IS*481*, the mPCR was repeated.

**Figure 2:**
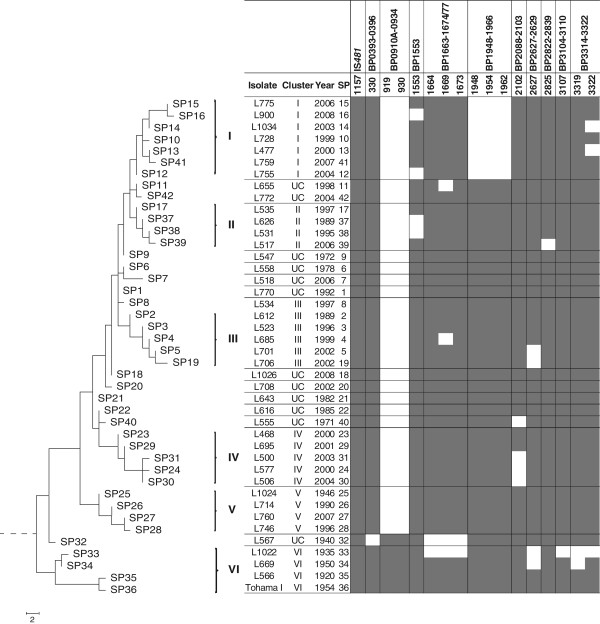
**Correlation between the losses of regions of difference (RDs) and single nucleotide polymorphism (SNP)-based evolution of** ***Bordetella pertussis*** **.** RDs which were variable across 42 *B. pertussis* isolates from unique SNP profiles (SP) were plotted against the evolution of *B. pertussis* as determined previously by SNP typing. Grey boxes indicate the presence of a gene whereas white boxes indicate the absence of a gene. In total 16 genes were variable, representing 10 RDs. Genes representing RDs which were present in all strains were not included in this Figure. Roman numerals represent *B. pertussis* clusters determined by SNP typing [14] and ‘UC’ denotes isolates that were not assigned to a specific SNP cluster.

In this study, multiple PCR targets were combined in an mPCR/RLB assay to simultaneously identify and differentiate *B. pertussis* isolates based on patterns of gene loss. An initial mPCR/RLB was performed using *B. pertussis* Tohama I because it is a completely sequenced and well-annotated historic isolate collected in 1954 [2] which has been often used as a vaccine production strain. Therefore, it was expected that all selected genes would be present. 32 genes combined into 5 multiplex reactions and representing 24 RDs are presented in Table 1. IS*481*, an insertion sequence commonly used for *B. pertussis* detection, was included in one of the mPCR as an internal positive control and was amplified in all isolates. The 42 isolates representing the SNP profiles of *B. pertussis* evolution were then typed using the 5-plex mPCR/RLB assay and the RDs for each isolate were recorded as present or absent. A representative blot showing positive signals and the absence of product binding is shown in Figure 1.Only 10 of the 24 RDs studied varied between isolates in the current set of 42. Two RDs were most frequently absent. BP0910A - BP00930 (represented by BP0919) was absent from the majority (37/42) of isolates and BP1948-BP1966 (represented by BP1948, BP1954 and BP1962) was absent from 7 of the 42 isolates. Other genes, which were absent from at least one isolate, were BP0330, BP1553, BP1664, BP1669, BP1673, BP2102, BP2627, BP2825, BP3107, BP3319, BP3322, which collectively represent 10 RDs (Figure 2). Not all genes within an RD were always absent. For example, two isolates (L655 and L685) had lost only one gene, BP1669 from BP1663-BP1674/77, whereas all three representative genes were absent from two other isolates (L1022 and L567) (Figure 2). Similarly, in in isolates L477, L1034 and L669 only one gene (either BP3319 or BP3322) from BP3314-BP3322 was absent, while both representative genes were lost from L1022.

**Table 1:** **Details of genes within selected regions of difference (RD) for this study and comparison to RD in other studies**

Genes within RDs*	Representative gene for this study	Gene function	King ***et al.***(2010) [5]	Brinig ***et al.***(2006) [16]	Heikkinen ***et al.***(2007) [19]	Caro ***et al.***(2006) [3]
BP0024- BP0030		MaoC family protein	RD1			
BP0393- BP0396		Hypothetical protein	RD2			
BP0502- BP0511		Hypothetical protein	RD3-RD4	RD1		
BP0513- BP0516	N/A					
BP0612- BP0644	N/A		RD6			
BP0593	N/A		RD7			
BP0712- BP0715	BP0711/BP0712	Putative phosopholipase	RD9	RD2		
BP0910A- BP0934	BP0919	Putative succinate-semialdehyde dehydrogenase [NADP+]	RD13	RD3	L1	RD-1
	BP0930	Putative CoA ligase				
BP1131-BP1141	BP1136	Heme uptake regulator	RD15-RD16	RD4-RD5	L2	RD-2
BP1158-BP1176	BP1170	Putative exported protein	RD17	RD6		RD-3
BP1225	N/A		RD19			
BP1553	BP1553	Putative exported protein	RD24			
BP1638-BP1639	BP1638	Hypothetical protein	RD25	RD7		
BP1663-BP1674/77	BP1664	Glutathione S-transferase	RD27	RD8		
	BP1669	Lactate dehydrogenase				
BP1676-BP1677	N/A		RD28	RD9		
BP1698	N/A		RD30			
BP1948-BP1966	BP1948	Branched-chain amino acid-binding protein	RD33	RD10	L3	RD-4
	BP1954	Probable oxidoreductase				
	BP1962	Putative ferrisiderophore receptor				
BP2088-BP2103	BP2102	lysR family transcriptional regulator	RD35		L4	
BP2133-BP2134	N/A		RD37			
BP2136-BP2139	N/A		RD38-RD39			RD-5
BP2167-BP2180	BP2167	Putative integral membrane protein	RD40			
BP2272-BP2274	BP2273	Putative periplasmic protein	RD41	RD11		RD-6
BP2517-BP2518	BP2518	Sarcosine oxidase beta subunit	RD43	RD12		
BP2519-BP2523	BP2522	FolD bifunctional protein	RD44			
BP2627-BP2629	BP2627	Pseudogene	RD45	RD13		RD-7
BP2670-BP2671	BP2671	Hypothetical protein	RD46	RD14		
BP2822-BP2839	BP2825	GntR family transcriptional regulator	RD48			
BP2883	N/A		-			RD-8
BP2921-BP2924	BP2921	Hypothetical protein	RD50	RD15		
BP3104-BP3110/3	BP3107	Putative gamma-glutamyltranspeptidase (exported protein)	RD52-RD54	RD16-RD17		RD-9
	BP3113	Pseudogene				
BP3188-BP3202	N/A		RD55			
BP3314-BP3322	BP3319	Putative IclR-family transcriptional regulator	RD56	RD18		RD-10
	BP3322	Putative binding-protein-dependent transport protein				
BP3352-BP3390	BP3384	Putative phage terminase	RD57-RD59	RD19		
BP3477	N/A		RD60			
BP3840-BP3861	BP3842	Hypothetical protein	RD63-RD64	RD20		
	BP3853	Conserved hypothetical protein				

*Gene nomenclature in this column is based on the numbering of genes in Tohama I (NC_002929).

N/A- RDs for which either primers or probes could not be selected for efficient mPCR/ RLB or which could not be amplified during mPCR and were subsequently left out of analysis.

### Relationships between RD absences and *B. pertussis*SNP evolution

To determine whether the presence of RD or absence was linked with the evolution of *B. pertussis*, the results of the mPCR/RLB assay were plotted against the SNP-based phylogenetic tree as previously determined by Octavia *et al.* [14] and shown in Figure 2. BP0910A- BP0930 was found only in isolates with SPs in cluster VI, which consists of isolates from the pre-vaccination era. In contrast, BP1948-BP1966 was detected in all isolates with SPs in all clusters except cluster I.

The association of these two RDs and SNP clusters could, potentially, be explained by carry-over (or hitchhiking) of gene losses in fitter *B. pertussis* strains [15]. Genetic hitchhiking, also known as selective sweeps, refer to genetic changes in neighboring genes that get fixed as a result of linkage with genes carrying advantageous mutations which are selected for, but these genetic changes may not necessarily contribute to the fitness of the organism. In *B. pertussis*, genetic hitchhiking is most clearly observed with the two RD losses. The loss of BP910A-BP930 is associated with the potentially advantageous change to *ptxA2* which arose when the whole cell vaccine was introduced, while the loss of BP1948-1966 is associated with changes to *prn2* and *ptxP3* in cluster I, which may be driven by selection pressure from the acellular vaccine as discussed previously [14]. The loss of BP1948-1966 may also be driven by immune selection pressure against BP1948 which encodes an immunogenic protein as discussed below.

Apart from these two major RD absences that correlated with specific SNP clustering, the majority of gene losses occurred independently in a ‘mosaic’ pattern, and were not associated with the introduction of vaccination or change in antigen alleles [16]. Even within clusters I and II, which are generally highly homogeneous, patterns of gene loss differed; BP1553 and BP3322 each absent from 2 of 7 isolates in cluster I and similarly, BP2825 and BP1553 absent from 1 and 2 isolates respectively, in cluster II.

A limitation of this study is that only single representatives of each SP were used and interpretation of sporadic gene losses from this collection of isolates is difficult without performing additional investigations with more isolates from SP clusters. However, in-depth analyses of French, Finnish, Dutch and Swedish *B. pertussis* isolates demonstrated that correlations could be also made between RD losses, specific pulse-field gel electrophoresis (PFGE) profiles and antigen alleles [3, 4, 17, 18]. The absence of BP1948-BP1966 in cluster I isolates in this study, in particular, can be likened to the same RD loss in Finnish isolates collected since 1999 which belong to PFGE Group IVβ [20]. As these isolates have only emerged recently, a strong temporal relationship with BP1948-BP1966 could be made. In addition, given the highly homogeneous nature of *B. pertussis* populations, it is likely that isolates within this PFGE profile are identical to cluster I isolates in this study. Previous reports of RD losses and virulence-associated alleles by King *et al.* [4, 5] showed a definitive relationship between BP1948-BP1966 loss and isolates carrying the *ptxP3* allele. All cluster I isolates in this study and additional isolates not included in this study carried the *ptxP3* allele in addition to *prn2*, a non-ACV allele [21]. Thus, the RD was lost in current, globally circulating isolates that have expanded over time from a single clone.

### Functional significance of gene losses from regions of difference

The RD encompassing BP910A-BP934 was absent from most isolates analysed by several other studies [4, 5, 19, 22] as well as this one. The defining feature of isolates, which had retained this RD, was the fact that all were collected before the introduction of whole cell vaccines. Within this RD, the majority of genes in BP910A-BP934 encode hypothetical proteins or transcriptional regulators. Genes of known function include a putative succinate-demialdehyde dehydrogenase and a citrate utilization protein.

The second major RD loss was BP1948-BP1966, which was absent from seven, all very recent, isolates of the 42 selected. RD BP1948-BP1966 is 22.7 kb in size and consists of 18 genes, the majority of which are involved in energy metabolism, transport or binding or are pseudogenes [3]. Interestingly, BP1948, which encodes a 44 kDa branched chain amino acid binding protein involved in membrane transport, has been identified as immunogenic by Tefon *et al.* and the deletion of this region may potentially be advantageous to *B. pertussis* if its loss decreases overall immunogenicity and herd immunity in a population [23].

Other genes of interest within variably absent RDs included copper resistance proteins within the RD BP3314-BP3322 (represented by BP3319 and BP3322) [16]. BP3322 was absent from three *B. pertussis* strains*,* two of which belong to the predominant SPs (SP13 and SP14) in SNP cluster I, whereas BP3319 was absent from L669 and both BP3319 and BP3322 were absent from L1022- two cluster VI strains. The regulation of copper concentrations is tightly controlled to prevent toxicity within the cell [3], although the exact effects of these gene losses have not been determined. The absence of the whole RD from earlier strains compared with individual gene losses from SP13 and SP14 indicate that some of these more recent losses occurred independently.

## Conclusions

The losses of RDs or RD-associated genes shown in this study demonstrate that genome changes, particularly genome reduction, are an ongoing process in *B. pertussis.* Most of the individual RD-associated gene losses have occurred randomly, except that the loss of two RDs was each shown to be temporally associated with an evolutionary event, as shown by a change in SNP cluster. However, these events are not likely to have been directly due to selection pressure but instead could be explained by “genetic hitchhiking” [15] in fitter *B. pertussis* variants as a result of selection somewhere else in the genome. We also demonstrated that an mPCR/RLB method can be used as a rapid method to detect presence or absence of RDs.

## Methods

### Bacterial isolates

Based on previous SNP typing of a diverse range of *B. pertussis* by Octavia *et al.* [14], one *B. pertussis* isolate from each of the 42 unique SNP profiles was selected to represent the evolution of *B. pertussis* since the 1920s. Where possible, isolates were selected to be most representative of their SP in terms of year and region of isolation. Bacterial isolates were grown on Bordet-Gengou agar (BD) supplemented with 10% defibrinated horse blood (Oxoid) for 3–5 days at 37°C. DNA was extracted using the phenol/chloroform method and used for mPCR.

### Identification and selection of regions of difference

RDs were previously defined by Cummings *et al.* [24] as two or more adjacent array elements not detected in at least 3 strains of *B. pertussis*. However, further studies identified additional RDs consisting of either single genes or two consecutive genes, which were variably present in different isolate collections [3–5, 16, 19] *.* For this study, previously identified RDs were considered suitable for analysis if there was at least one gene, which had been shown to be variably absent from previously studied *B. pertussis* populations. The prevalence of each RD loss was also calculated and RDs present in more than 98% of the *B. pertussis* population from each study were not studied. To avoid confusion, specific RDs are referred to in this study by the corresponding genes on either side of the RDs. A total of 35 RDs met the criteria and were used for further analysis. Larger RDs and RDs which had been shown to be most variable, had more than one gene selected to represent the RD. Six RDs met these criteria and therefore had more than one representative gene (Table 1).

### Primer and probe design

*B. pertussis* Tohama I, a completely sequenced reference strain (NC_002929) [2], was used as the basis for designing PCR primers and probes for each of the selected RDs. All primers were 18–24 bp in length and amplified regions with similar G + C content for efficient multiplexing. Primer interactions and dimer formation within mPCRs were analysed using Autodimer software [25]. Amplicons were 150–300 bp with annealing temperatures between 50-60°C, from regions within selected RDs, in order to allow simultaneous amplification in a multiplex reaction.

Corresponding RLB probes for each of the selected genes were also designed using Tohama I genome as a reference to ensure specific binding. Each probe was designed to be 20–22 bp in length and complementary to individual PCR products. Each probe was labelled at the 5’ end with an amine group to allow binding to the nylon membrane. The sequences of forward and reverse primers, expected PCR product size, corresponding RD probes and the function of each gene of interest are listed in Additional file 1: Table S1. The selected genes from each RD were each individually tested by single-plex PCR on a subset of strains and confirmed on 2% agarose gel to determine whether the selected regions/genes were absent before combining primer sets into a multiplex-PCR.

### Multiplex PCR

Each mPCR was optimised to contain at least 8–9 targets as it had not been possible to include all targets in one mPCR. Each reaction mixture consisted of 8–9 primer pairs at a concentration of 10 μmol each, ~30 ng genomic DNA, 2U Taq polymerase (Biotium), 0.25 mM each of dATP, dTTP, dGTP (Bioline) and 0.125 mM each of biotinylated/non-biotinylated dCTP (Roche), 3.5 mM MgCl_2_ and 1 M betaine (Sigma-Aldrich). Thermocycling conditions included a touchdown step and were as follows: initial denaturation at 96°C for 2 min; 35 cycles of 96°C for 30 sec, 55°C for 30 sec, 72°C for 15 sec; and final extension at 72°C for 7 min. Individual PCR confirmation was then carried out to determine the reliability of mPCR in amplifying desired products and whether any mis-amplification or primer dimer formation occurred.

### Reverse line blot assay

The reverse line blot assay was carried out according to Kong *et al.* [6]. Briefly, a Biodyne C membrane was activated with 16% (w/v) 1-ethyl-3-(3-dimethylaminopropyl) carbodiimide (EDAC) for 20 min and rinsed with sterile milliQ water. The membrane was placed in a Miniblotter® (Immunetics) and specific 5’ amine labeled oligonucleotide probes were then bound to the membrane and fixed with 0.1 M NaOH. The membrane was then removed, rotated 90° and placed back into the Miniblotter. Products from the five mPCR were combined and boiled for 5 min before being applied to the membrane perpendicular to the bound probes. The membrane was then incubated at 55°C for 1 hr before washing in 1× SSPE (150 mM sodium chloride, 10 mM sodium phosphate and 1 mM EDTA)/0.1% SDS solution. A streptavidin-peroxidase conjugate (Roche) was then added and the membrane further incubated at 42°C for 1 hr before washing and exposing with enhanced chemiluminescence (ECL) detection kit (GE LifeSciences). Chemiluminescence was detected using LAS3000 Imager (Fujifilm).

A clear hybridisation signal was interpreted as the presence of the corresponding gene, whereas the lack of signal was interpreted as the absence of a gene. Faint or indistinct hybridisations deemed ambiguous were confirmed using individual PCR and agarose gel electrophoresis.

### Ethics statement

This work did not involve human subjects, human material or human data and therefore did not require ethical approval.

## Electronic supplementary material

Additional file 1: Table S1: **Primers and probes and used in this study.** (XLSX 16 KB)
